# Proteins that contain a functional Z-DNA-binding domain localize to cytoplasmic stress granules

**DOI:** 10.1093/nar/gkt750

**Published:** 2013-08-27

**Authors:** Siew Kit Ng, Rebekka Weissbach, George E. Ronson, A. D. J. Scadden

**Affiliations:** Department of Biochemistry, University of Cambridge, Cambridge CB2 1QW, UK

## Abstract

Long double-stranded RNA may undergo hyper-editing by adenosine deaminases that act on RNA (ADARs), where up to 50% of adenosine residues may be converted to inosine. However, although numerous RNAs may undergo hyper-editing, the role for inosine-containing hyper-edited double-stranded RNA in cells is poorly understood. Nevertheless, editing plays a critical role in mammalian cells, as highlighted by the analysis of ADAR-null mutants. In particular, the long form of ADAR1 (ADAR1^p150^) is essential for viability. Moreover, a number of studies have implicated ADAR1^p150^ in various stress pathways. We have previously shown that ADAR1^p150^ localized to cytoplasmic stress granules in HeLa cells following either oxidative or interferon-induced stress. Here, we show that the Z-DNA-binding domain (Zα^ADAR1^) exclusively found in ADAR1^p150^ is required for its localization to stress granules. Moreover, we show that fusion of Zα^ADAR1^ to either green fluorescent protein (GFP) or polypyrimidine binding protein 4 (PTB4) also results in their localization to stress granules. We additionally show that the Zα domain from other Z-DNA-binding proteins (ZBP1, E3L) is likewise sufficient for localization to stress granules. Finally, we show that Z-RNA or Z-DNA binding is important for stress granule localization. We have thus identified a novel role for Z-DNA-binding domains in mammalian cells.

## INTRODUCTION

Adenosine deaminases that act on RNA (ADARs) catalyze the conversion of adenosine (A) to inosine (I) within double-stranded RNA (dsRNA). Three ADARs have been described in mammalian cells (ADARs 1–3), although only ADAR1 and ADAR2 appear to be catalytically active (1,2).

Although a single gene encodes ADAR1, two different isoforms are generated by use of alternative promoters and alternative splicing (3,4). The short form of ADAR1 (ADAR1 p110; ADAR1^p110^) is constitutively expressed and localizes mainly to the nucleus, whereas the long form of ADAR1 (ADAR1p150; ADAR1^p150^) is interferon-inducible and shuttles between the nucleus and cytoplasm. However, the localization of ADAR1^p150^ is predominantly cytoplasmic (3–7). With the exception of the N-terminal region, where ADAR1^p110^ is truncated by 295 amino acids relative to ADAR1^p150^, the two proteins are otherwise identical (1,3,8). Both proteins have in common three dsRNA-binding domains (dsRBDs) and a C-terminal deaminase domain. The proteins differ in that ADAR1^p150^ has two Z-DNA/Z-RNA-binding domains (ZBDs; Zα and Zβ) within the N-terminal region, whereas only the Zβ domain is present in the N-terminally truncated ADAR1^p110^ (9). This difference will have functional consequences, as only the Zα domain has the ability to bind Z-DNA/Z-RNA (10). Recent *in vitro* studies have shown that binding of ADAR1^p150^ to a Z-RNA-forming motif enhances editing within adjacent A-form dsRNA sequences. The Zα domain may thus be used to target ADAR1^p150^ to sequences with the potential to form Z-RNA for selective editing (11).

Z-DNA or Z-RNA comprises an alternative, higher energy conformation of DNA or RNA, respectively, that preferentially occurs within sequences containing alternating purine and pyrimidine residues to give a characteristic zigzag helix (12). Although transition to the higher-order structure is energetically unfavorable, proteins containing Z-DNA-binding domains can specifically induce and stabilize both Z-DNA and Z-RNA. Moreover, structural analyses have revealed that recognition and binding of both Z-DNA and Z-RNA by Zα domains is conformation-specific rather than sequence-specific (10,13,14). As the left-handed Z-conformations of Z-DNA and Z-RNA are similar, the Zα domain may thus represent an unusual class of domains that specifically bind to both duplex DNA and RNA (15).

In addition to ADAR1^p150^, Z-DNA/Z-RNA-binding domains have been identified in only a limited number of proteins. These include the Z-DNA-binding protein 1 [ZBP1; also known as DLM-1 or DAI (DNA-dependent activator of IFN-regulatory factors)] (14,16), the poxvirus virulence factor E3L (17,18), a protein kinase (PKZ) that was uniquely identified in fish (19) and a protein (ORF112) from the family *Alloherpesviridae*, which includes important pathogens of fish and amphibian species (20). Intriguingly, all of these proteins play a role in immune pathways. ADAR1^p150^, ZBP1 and PKZ are upregulated by interferon and are implicated in host defense mechanisms (3,4,16,19,21,22). In contrast, the Zα domain of the E3L protein is required for viral pathogenicity (22). It is possible that the Zα domain of E3L competes with cellular Z-DNA-binding proteins for binding of specific target sequences, and thus subverts the host interferon response (23). The Zα domain of ORF112 similarly has the potential to antagonize PKZ function in fish by binding its cellular targets (20).

We have recently shown that ADAR1^p150^ localizes to cytoplasmic stress granules (24). Stress granules are cytoplasmic foci that provide eukaryotic cells a means of survival during stress (25–27). Stress granules typically form as the result of translational inhibition and therefore comprise translationally arrested mRNA as well as stalled initiation complexes and small ribosomal proteins (28). Importantly, sequestration of a set of translationally arrested mRNAs allows selective synthesis of proteins required for cell survival. When the stress conditions are removed, mRNAs sequestered within stress granules may reassemble on polysomes and resume translation (25). Alternatively, mRNAs may be degraded within cytoplasmic-processing bodies (P-bodies), which are dynamically linked to stress granules (29).

Here, we present experimental data showing that the Zα domain of ADAR1^p150^ is necessary and sufficient for localization of ADAR1^p150^ to stress granules. Fusion of the Zα domain to either green fluorescent protein (GFP) or polypyrimidine tract binding protein 4 (PTB4) resulted in their localization to stress granules. Moreover, we demonstrate that the Zα domain from ZBP1 and E3L also direct localization to stress granules. Finally, we provide evidence that binding of the Zα domain to Z-RNA/Z-DNA is important for localization of ADAR1^p150^ to stress granules.

## MATERIALS AND METHODS

### Transfections

HeLa cells were maintained in DMEM with GlutaMAX™-I (Gibco) supplemented with 10% (v/v) fetal bovine serum. HeLa cells (2 × 10^5^ cells per well (6 well plate)) were used for transfections. Plasmids were transfected into HeLa cells using Lipofectamine-2000 (LF-2000; Invitrogen), and cells visualized after 24 h. Poly(IC) (10 ng; Sigma) was typically used to transfect HeLa cells using LF-2000, and cells were visualized after 7 h. Mock transfections used LF-2000 alone. For arsenite treatment, cells were incubated in media supplemented with 0.5 mM sodium arsenite (Sigma) for 30 min, then allowed to recover for 30 min in the absence of arsenite (28). Stress granules were identified in HeLa cells with reference to the stress granule marker TIAR (red). As the localization of TIAR in untreated cells is predominantly nuclear, stress granules were readily identifiable as distinct cytoplasmic foci. GFP-tagged or Flag-tagged proteins (green) were then analyzed for co-localization with TIAR in stress granules. To determine the efficiency of localization of ADAR1^p150^ (or other constructs) to stress granules, at least 100 cells positive for the stress granule marker TIAR were analyzed for co-localization of ADAR1^p150^ (*n* ≥ 3; error bars are mean ± SD). The *t*-tests (two-tailed, unequal variance) were used. Cell lysates were prepared using NET buffer (50 mM Tris-HCl, pH 7.5, 150 mM NaCl, 0.5% [v/v] NP40, 1 mM EDTA), and protein concentrations were determined by Bradford assays.

### Expression constructs

GFP-ADAR1 p150 (GFP-Adar1^p150^) was as described previously (6). Other constructs were prepared using standard cloning procedures (30). For GFP-ADAR1^p110^, the ADAR1^p110^ sequence [amino acids (aa) 296–1226; numbered as for ADAR1^p150^] was amplified by PCR using primers containing *Xho*1 and *Hin*dIII restriction sites, and inserted into pEGFP-C1 (Clontech). The same strategy was used to clone the following ADAR1 domains into pEGFP-C1: Z (aa 1–361), ZΔ (aa 1–295), ZR (aa 1–807), R (aa 486–807), RD (aa 486–1226), D (aa 812–1226), and Zα^ADAR1^ (aa 123–209). For Flag-ADAR1^p150^, full-length ADAR1 (aa 1–1226) was amplified by PCR using primers containing *Hin*dIII and *Xba*I restriction sites, and inserted into p3xFLAG-CMV™-7.1 (Sigma). The same strategy was used to clone Flag-ADAR1^p110^ (aa 296–1226) and Flag-ZΔ (aa 1–295). For Flag-ΔN-NES-ADAR1, the oligonucleotide sequence 5′- GGCAGAGAGGTGTTGATTGCCTTTCCTCACATTTCCAGGAACTGAGTATCTACGAA was cloned into the *Sac*II and *Bgl*II sites within Flag-ADAR1^p150^. Mutations within Flag-ADAR1^p150^ (K169A, E171A, Y177A, E912A) were introduced by site-directed mutagenesis (Stratagene). For GFP-Zα^ZBP1^, the Zα domain from ZBP1 (aa 4–85) was amplified by PCR using primers containing *Xho*1 and *Hin*dIII restriction sites, and inserted into pEGFP-C1 (Clontech). ZBP1-GFP was as described previously (23). For Flag-PTB4-Zα^ADAR1^ and Flag-Zα^ADAR1^-PTB4, the Zα^ADAR1^ domain (aa 123–209) was initially amplified by PCR using primers with *Eco*RI and *Bgl*II restriction sites, and inserted into p3xFLAG-CMV™-7.1. The inclusion or exclusion of a stop codon following Zα^ADAR1^ gave rise to Flag-Zα^ADAR1^-stop or Flag-Zα^ADAR1^, respectively. Sequences corresponding to PTB4 (aa 1–557) were subsequently amplified by PCR using primers containing *Hin*dIII and *Eco*RI restriction sites, and inserted into Flag-Zα^ADAR1^-stop to give Flag-PTB4-Zα^ADAR1^. Alternatively, the sequence corresponding to PTB4 was amplified by PCR using primers containing *Bgl*II and *Sal*I restriction sites, and inserted into Flag-Zα^ADAR1^ to give Flag-Zα^ADAR1^-PTB4. Flag-PTB4 was as described previously (31,32). For GFP-E3L, the E3L gene from vaccinia virus (Western Reserve strain) (aa 1–190) was amplified from genomic DNA by PCR using primers with *Xho*I and *Hin*dIII restriction sites, and inserted into pEGFP-C1 (Clontech). For GFP-Zα^E3L^, the Zα domain from E3L (aa 1–83) was amplified by PCR using primers containing *Xho*1 and *Hin*dIII restriction sites, and inserted into pEGFP-C1 (Clontech). All constructs were confirmed by sequencing.

### Immunofluorescence

HeLa cells were plated on 13 mm glass cover slips in six-well plates, and grown for 24 h (cells were 50–70% confluent). Cells were washed in phosphate buffered saline (PBS), incubated 20 min in 4% (v/v) paraformaldehyde and then rewashed in PBS. Cells were permeabilized in 0.5% Triton-X (Fisher Scientific) for 3 min, washed with PBS, then incubated in blocking buffer (1% (w/v) BSA in PBS) for 1 h. Cells were subsequently incubated for 1 h with primary antibodies diluted in blocking buffer, washed extensively with PBS and then incubated with secondary antibodies for 1 h. Primary antibodies used were TIAR (Sc-1749; Santa Cruz Biotech, Inc.) and Flag (F3165; Sigma). Secondary antibodies were labeled with either Rhodamine Red-X RRX (red) or DyLight 488 or Alexa Fluor 488 (green) (Jackson ImmunoResearch). Cells were washed three times with PBS, dried and then mounted in ProLong® Gold antifade reagent with DAPI (Invitrogen). Cells were viewed and photographed using a Zeiss Axioimager M1 microscope (Carl Zeiss MicroImaging). Images were compiled using Adobe Photoshop.

### Immunoblots

Proteins (50 μg) were separated by SDS–PAGE, transferred to PVDF and detected using enhanced chemiluminescence (ECL). Antibodies were GFP (sc-9996; Santa Cruz Biotech, Inc.); Flag (F3165; Sigma) and actin (A2066; Sigma). Secondary antibodies were horseradish peroxidase coupled (Jackson ImmunoResearch).

## RESULTS

We have previously shown that ADAR1^p150^ localizes to cytoplasmic stress granules in HeLa cells during either arsenite-induced oxidative stress or following transfection of long dsRNA (24). We now aimed to further characterize the localization of ADAR1^p150^ in stress granules.

### Localization of ADAR1^p150^ to stress granules does not require deaminase activity

We previously considered mechanisms by which ADAR1^p150^ may be recruited to cytoplasmic stress granules (24). One possible explanation was that ADAR1^p150^ localized to stress granules by virtue of association with IU-dsRNA following editing of cytoplasmic RNA targets. This idea was based on previous data that showed that IU-dsRNA bound to a ‘stress-granule-like complex’ that may precede stress granule assembly (33). We thus asked whether a functional deaminase domain of ADAR1^p150^ was required for its localization to stress granules.

**Figure 1.**
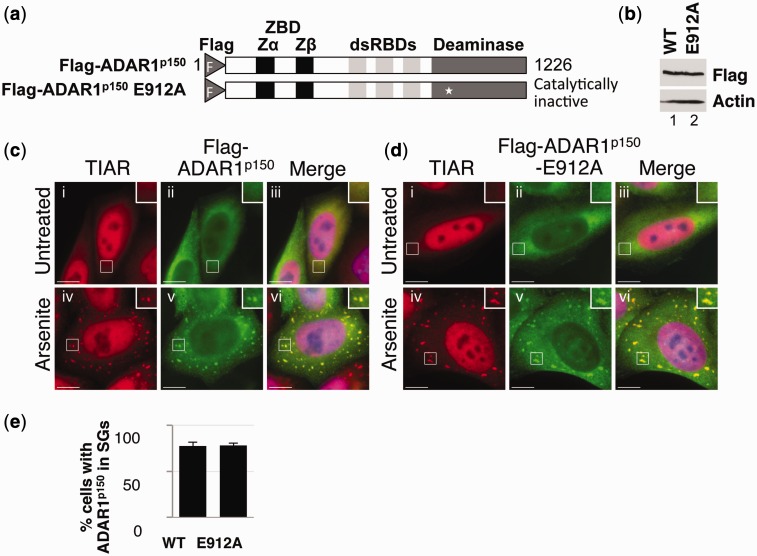
Deaminase activity is not required for stress granule localization. (**a**) A schematic diagram of Flag-ADAR1^p150^ and Flag-ADAR1^p150^ E912A. The white star indicates the position of the point mutation in Flag-ADAR1^p150^ E912A. The Flag epitope tag (F), Z-DNA-binding domains (ZBD; Zα and Zβ), dsRBDs and deaminase domain are indicated. (**b**) HeLa cells were transfected with expression vectors for wild-type Flag-ADAR1^p150^ (WT) and Flag-ADAR1^p150^ E912A (E912A), and lysates prepared after 24 h. Immunoblotting with a Flag antibody was used to analyze expression. Actin was a loading control. (**c** and **d**) HeLa cells transiently transfected with expression vectors for Flag-ADAR1^p150^ (c) and Flag-ADAR1^p150^ E912A (d). After 24 h, the cells were cultured in the absence (i–iii) or presence of arsenite (iv–vi) before processing for visualization of TIAR (red; i and iv) or Flag-tagged proteins (green; ii and v) using fluorescence microscopy. DAPI staining is in blue (Merge; iii and vi). Bar = 10 μm. (**e**) TIAR was used as a marker to identify stress granule-containing cells, and the proportion of cells with stress granules positive for wild-type Flag-ADAR1^p150^ (WT) or Flag-ADAR1^p150^ E912A (E912A) was then determined. Error bars are mean ± SD (*n* = 3).

Expression constructs were prepared which encoded Flag-tagged full-length ADAR1^p150^ (Flag-ADAR1^p150^) or a Flag-tagged catalytically inactive mutant of ADAR1^p150^ (Flag-ADAR1^p150^ E912A), as described previously (34) (Figure 1a). HeLa cells were then transfected with Flag-ADAR1^p150^ and Flag-ADAR1^p150^ E912A, and after 24 h, the cells were cultured in the absence or presence of arsenite. Immunoblotting using a Flag antibody confirmed that expression was similar for each construct (Figure 1b). HeLa cells were subsequently fixed and stained with antibodies specific for TIA-1 related protein (TIAR), a stress granule marker (red) or Flag (green) and visualized using fluorescent microscopy. DAPI (blue) was used to stain the nucleus in this and all subsequent experiments. In the absence of arsenite (Untreated), TIAR localized to both the nucleus and cytoplasm, although was predominantly nuclear (Figure 1c and d; i and iii). In contrast, Flag-ADAR1^p150^ and Flag-ADAR1^p150^ E912A were largely cytoplasmic (Figure 1c and d; ii and iii). This is consistent with previous observations (6,24). Following arsenite treatment (Arsenite), TIAR, Flag-ADAR1^p150^ and Flag-ADAR1^p150^ E912A co-localized to stress granules (Figure 1c and d; iv–vi). The relative efficiency of localization of Flag-ADAR1^p150^ and Flag-ADAR1^p150^ E912A to stress granules was subsequently determined, which showed that Flag-ADAR1^p150^ and Flag-ADAR1^p150^ E912A localized to cytoplasmic stress granules with comparable efficiencies (Figure 1e). Together, these data showed that a functional deaminase domain was not required for localization of ADAR1^p150^ to stress granules.

### ADAR1^p150^ constructs that contained the Zα domain localized to stress granules

Localization of ADAR1^p150^ to stress granules occurred independently of a functional deaminase domain (Figure 1). We next undertook experiments that aimed to understand how ADAR1^p150^ was localized to stress granules. Initially, we asked which domains of ADAR1^p150^ were responsible for its localization to stress granules.

**Figure 2.**
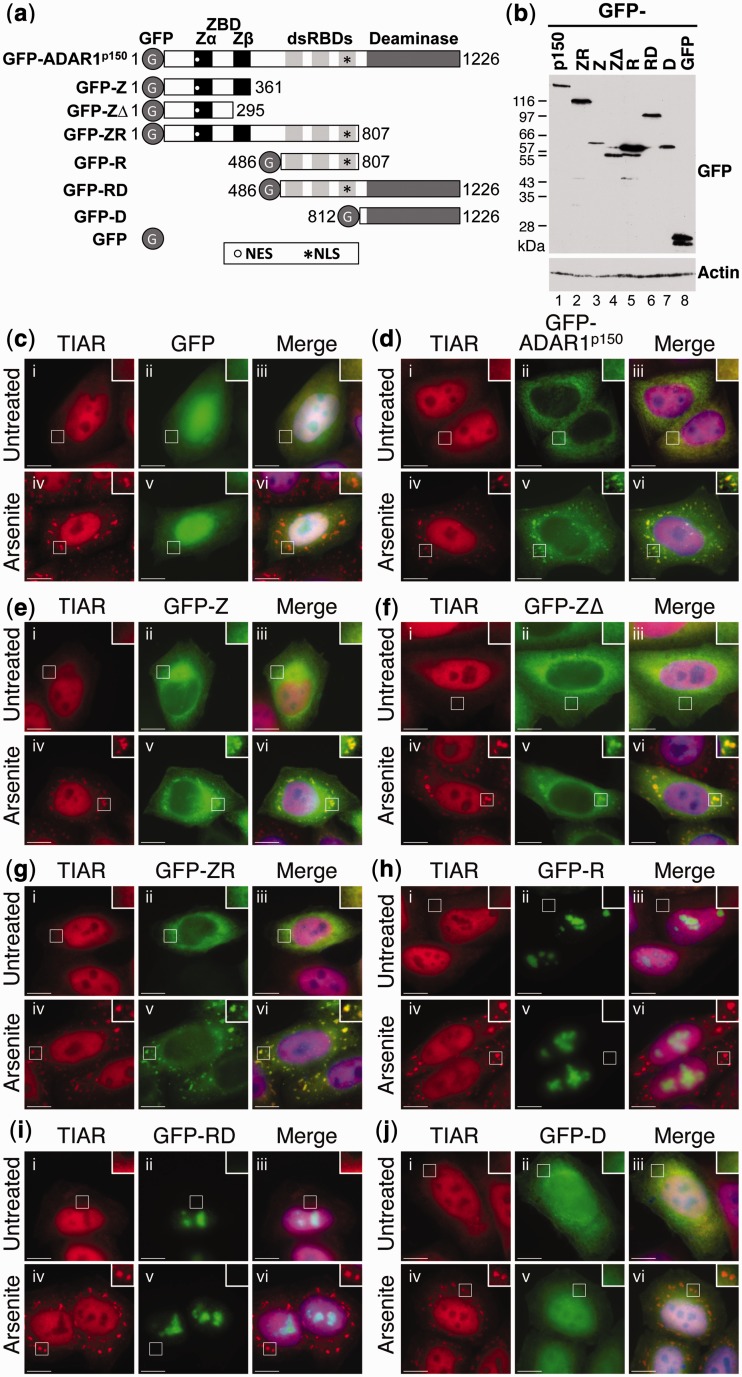
The Zα domain of ADAR1^p150^ is required for localization to stress granules. (**a**) A schematic diagram of the GFP-ADAR1^p150^ constructs; the amino acids of ADAR1^p150^ included in each construct are shown. The position of the NES and NLS in each construct is shown as a white circle and a black asterisk, respectively. GFP (G), the Z-DNA-binding domains (ZBD; Zα and Zβ), dsRBDs and deaminase domain are indicated. (**b**) HeLa cells were transfected with expression vectors for GFP-ADAR1^p150^ (p150), GFP-ZR, GFP-Z, GFP-ZΔ, GFP-R, GFP-RD, GFP-D or GFP, and lysates were prepared after 24 h. Immunoblotting with a GFP antibody was used to analyze expression. Actin was a loading control. (**c–j**) HeLa cells were transiently transfected with expression vectors for GFP (c), GFP-ADAR1^p150^ (d), GFP-Z (e), GFP-ZΔ (f), GFP-ZR (g), GFP-R (h), GFP-RD (i) and GFP-D (j). After 24 h, the cells were cultured in the absence (i–iii) or presence of arsenite (iv–vi) before processing for visualization of TIAR (red; i and iv) or GFP-tagged proteins (green; ii and v) using fluorescence microscopy. DAPI staining is in blue (Merge; iii and vi). Bar = 10 μm.

Expression constructs were prepared where GFP was fused to various domains of ADAR1^p150^ (Figure 2a), where the domain boundaries were in accordance with those used in previous studies (21,35). HeLa cells were subsequently transfected with the various ADAR1 expression constructs (GFP-ADAR1^p150^, GFP-Z, GFP-ZΔ, GFP-ZR, GFP-R, GFP-D) or with a control construct expressing GFP alone (GFP), and after 24 h, the cells were cultured in the absence (Untreated) or presence of arsenite (Arsenite). Immunoblotting using a GFP antibody confirmed that expression was similar for each construct (Figure 2b). HeLa cells were subsequently fixed and processed for visualization of TIAR (red) and the GFP-tagged proteins (green) using fluorescent microscopy. Although the GFP-tagged proteins could be visualized directly, specific antibodies were used to detect TIAR. In the absence of arsenite treatment, GFP and GFP-D co-localized with TIAR in both nuclear and cytoplasmic compartments, although they were substantially enriched in the nucleus (Figure 2c and j, respectively; i–iii). In contrast, GFP-ADAR1^p150^, GFP-Z, GFP-ZΔ and GFP-ZR were largely cytoplasmic in untreated cells (Figure 2d–g; ii and iii). Finally, GFP-R and GFP-RD appeared to localize exclusively to the nucleus, where they were found predominantly in the nucleolus (Figure 2h; i, ii and iii). The observed distribution of the ADAR1 domains was consistent with the position of nuclear export and import signals previously identified (Figure 2a). A nuclear export signal (NES) is present within the Zα domain of ADAR1^p150^, whreas a nuclear localization signal (NLS) is located within the third dsRBD of ADAR1 (5,7). Thus, although GFP-ADAR1^p150^, GFP-Z, GFP-ZΔ and GFP-ZR contained a NES and were localized to the cytoplasm, its absence in GFP-R and GFP-RD resulted in nuclear localization. Following treatment of HeLa cells with arsenite, GFP-ADAR1^p150^, GFP-Z, GFP-ZΔ and GFP-ZR co-localized with TIAR in stress granules (Figures 2d–g; iv–vi). In contrast, GFP-R, GFP-RD and GFP-D were not detected in stress granules following arsenite treatment (Figures 2h–j; iv–vi). Equivalent observations were made using Flag-tagged ADAR1 domains (data not shown). Analysis of these data together revealed that the only domain common to all of the ADAR1 constructs that localized to stress granules was the Zα domain. We therefore speculated that the N-terminal Zα domain was responsible for localization of ADAR1^p150^ to stress granules.

### ADAR1^p110^ does not localize to stress granules

**Figure 3.**
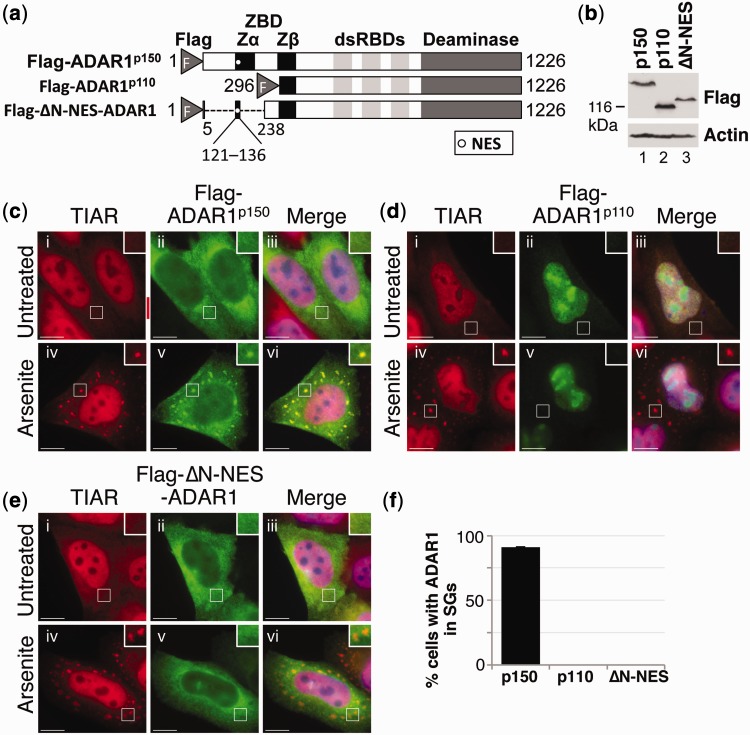
ADAR1^p110^ does not localize to stress granules. (**a**) A schematic diagram of Flag-ADAR1^p150^, Flag-ADAR1^p110^ and Flag-ΔN-NES-ADAR1. The position of the NES is shown as a white circle. The Flag epitope tag (F), Z-DNA-binding domains (ZBD; Zα and Zβ), dsRBDs and deaminase domain are indicated. (**b**) HeLa cells were transfected with the expression constructs Flag-ADAR1^p150^ (p150), Flag-ADAR1^p110^ (p110) or Flag-ΔN-NES-ADAR1 (ΔN-NES), and lysates are prepared after 24 h. Immunoblotting with a Flag antibody was used to analyze expression. Actin was a loading control. (**c–e**) HeLa cells were transiently transfected with expression vectors for Flag-ADAR1^p150^ (c), Flag-ADAR1^p110^ (d) and Flag-ΔN-NES-ADAR1 (e). After 24 h, the cells were cultured in the absence (i–iii) or presence of arsenite (iv–vi) before processing for visualization of TIAR (red; i and iv) or Flag-tagged proteins (green; ii and v) using fluorescence microscopy. DAPI staining is in blue (Merge; iii and vi). Bar = 10 μm. (**f**) TIAR was used as a marker to identify stress granule-containing cells, and the proportion of cells with stress granules positive for Flag-ADAR1^p150^ (p150), Flag-ADAR1^p110^ (p110) or Flag-ΔN-NES-ADAR1 (ΔN-NES) was then determined. Error bars are mean ± SD (*n* = 3).

As described earlier, ADAR1^p110^ is truncated at the N-terminus by 295 amino acids, relative to full-length ADAR1^p150^. Importantly, this truncation effectively removes the Zα domain, although the Zβ domain remains intact (Figure 3a). We thus investigated whether ADAR1^p110^ localized to stress granules in the absence of the Zα domain.

HeLa cells were transfected with expression constructs encoding Flag-ADAR1^p150^ and Flag-ADAR1^p110^ (Figure 3a), and after 24 h, they were cultured in the absence or presence of arsenite. Immunoblotting using a Flag antibody confirmed that expression of Flag-ADAR1^p150^ and Flag-ADAR1^p110^ was similar (Figure 3b). Cells were subsequently fixed and stained with antibodies specific for TIAR (red) or Flag (green) and visualized using fluorescent microscopy. In the absence of arsenite, ADAR1^p150^ was predominantly cytoplasmic while ADAR1^p110^ was found exclusively in the nucleus, where it was enriched in the nucleoli (Figure 3c and d; ii and iii). Nuclear localization of ADAR1^p110^ was due to the absence of the NES located within the Zα domain of ADAR1^p150^ (Figure 3a), as reported previously (5,6). When HeLa cells were treated with arsenite, ADAR1^p150^ co-localized with TIAR in cytoplasmic stress granules (Figure 3c; iv–vi). In contrast, ADAR1^p110^ remained in the nucleus and did not localize to stress granules following arsenite treatment (Figure 3d; iv–vi). These data are therefore in keeping with the idea that the Zα domain is required for localization to stress granules. On the other hand, an alternative explanation was that ADAR1^p110^ was not recruited to stress granules due to its nuclear localization. However, this seemed unlikely as other nuclear proteins re-localize to stress granules during stress (e.g. HuR, TIAR) (25). Nevertheless, to address this possibility, a new construct was prepared in which a deletion was made within Flag-ADAR1^p150^ that essentially removed the majority of the N-terminal region that is not present in ADAR1^p110^ (aa 6–237), while retaining the short sequence that contains the NES (aa 121–136), giving rise to Flag-ΔN-NES-ADAR1 (Figure 3a). HeLa cells were subsequently transfected with Flag-ΔN-NES-ADAR1, and after 24 h, they were cultured in the absence or presence of arsenite. Expression of Flag-ΔN-NES-ADAR1 (ΔN-NES) was verified using immunoblotting (Figure 3b). Cells were then fixed and stained with TIAR (red) or Flag (green) antibodies and visualized using fluorescent microscopy. In the absence of arsenite, Flag-ΔN-NES-ADAR1 was found predominantly in the cytoplasm (Figure 3e; ii and iii). Retention of the N-terminal NES was therefore effective in directing nuclear export, in contrast to what was seen with Flag-ADAR1^p110^ (compare Figure 3e and d; ii). When HeLa cells were treated with arsenite, TIAR localized to stress granules as expected (Figure 3e; iv). In contrast, Flag-ΔN-NES-ADAR1 did not co-localize with TIAR but remained distributed throughout the cytoplasm (Figure 3e; iv–vi). These data therefore demonstrated that despite its cytoplasmic localization, Flag-ΔN-NES-ADAR1, which was largely equivalent to Flag-ADAR1^p110^, did not localize to stress granules. Importantly, these data supported the idea that the Zα domain is required for localization to stress granules.

### The Zα domain is sufficient for stress granule localization

The data described in Figures 2 and 3 suggested that the Zα domain is required for localization of ADAR1 to stress granules. We next directly tested whether the minimal Zα domain from ADAR1^p150^ (Zα^ADAR1^) was sufficient for localization of proteins to stress granules.

**Figure 4.**
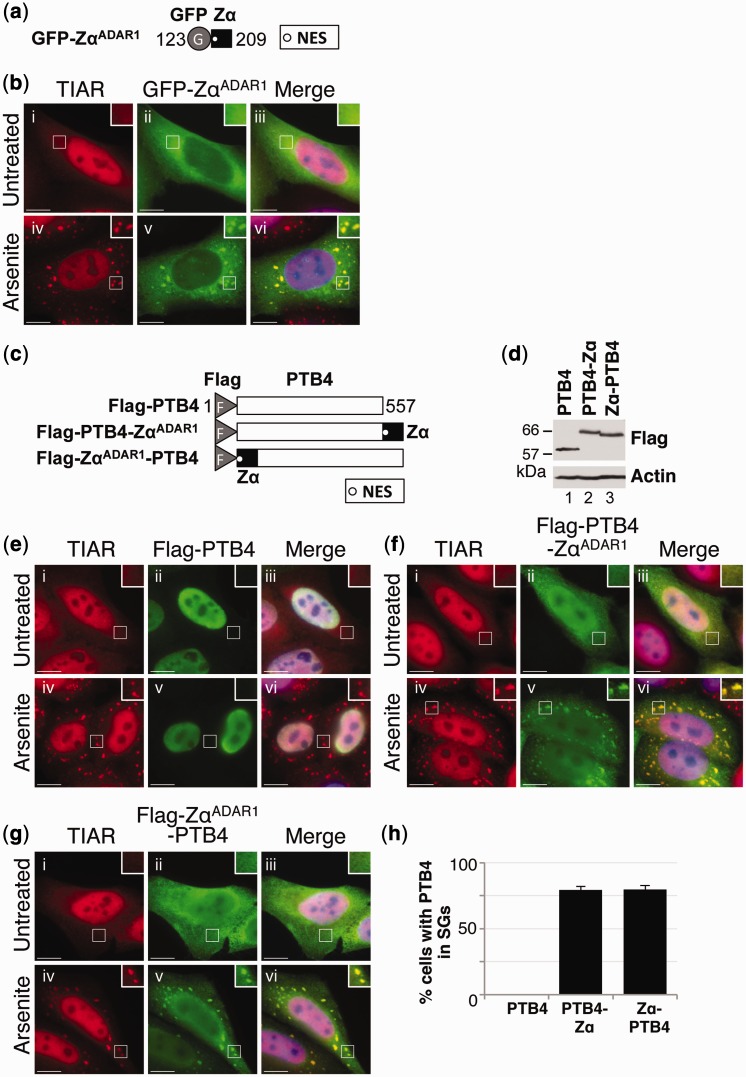
The Zα domain of ADAR1^p150^ is sufficient for localization to stress granules. (**a**) A schematic diagram of GFP-Zα^ADAR1^. The position of the NES is shown as a white circle. The Flag epitope tag (F) and Zα domain are indicated. (**b**) HeLa cells were transiently transfected with the expression vector GFP-Zα^ADAR1^. After 24 h, the cells were cultured in the absence (i–iii) or presence of arsenite (iv–vi) before processing for visualization of TIAR (red; i and iv) or GFP-Zα^ADAR1^ (green; ii and v) using fluorescence microscopy. DAPI staining is in blue (Merge; iii and vi). Bar = 10 μm. (**c**) A schematic diagram of Flag-PTB4, Flag-PTB4-Zα^ADAR1^ and Flag-Zα^ADAR1^-PTB4. The position of the NES is shown as a white circle. The Flag epitope tag (F) and Zα domains are indicated. (**d**) HeLa cells were transiently transfected with Flag-PTB4 (PTB4), Flag-PTB4-Zα^ADAR1^ (PTB4-Zα) and Flag-Zα^ADAR1^-PTB4 (Zα-PTB4), and lysates were prepared after 24 h. Immunoblotting with a Flag antibody was used to analyze expression. Actin was a loading control. (**e–g**) HeLa cells were transiently transfected with expression vectors for Flag-PTB4. (e) Flag-PTB4-Zα^ADAR1^ (f) and Flag-Zα^ADAR1^-PTB4 (g). After 24 h, the cells were cultured in the absence (i–iii) or presence of arsenite (iv–vi) before processing for visualization of TIAR (red; i, iv) or Flag-tagged proteins (green; ii and v) using fluorescence microscopy. DAPI staining is in blue (Merge; iii and vi). Bar = 10 μm. (**h**) TIAR was used as a marker to identify stress granule-containing cells, and the proportion of cells with stress granules positive for Flag-PTB4 (PTB4), Flag-PTB4-Zα^ADAR1^ (PTB4-Zα) or Flag-Zα^ADAR1^-PTB4 (Zα-PTB4) was then determined. Error bars are mean ± SD (*n* = 3).

An expression construct was prepared in which the short sequence encoding the Zα^ADAR1^ domain was placed downstream of the coding region for GFP (Figure 4a; GFP-Zα^ADAR1^). The Zα^ADAR1^ domain boundaries chosen were based on those used in previous studies (13,15,36). HeLa cells were subsequently transfected with GFP-Zα^ADAR1^, and after 24 h, they were cultured in the absence or presence of arsenite. Immunoblotting confirmed that GFP-Zα^ADAR1^ was expressed at a level comparable with GFP-ADAR1^p150^ (data not shown). Cells were subsequently fixed and processed for visualization of TIAR (red) and GFP-Zα^ADAR1^ (green) using fluorescence microscopy. In the absence of arsenite, TIAR was predominantly nuclear, whereas GFP-Zα^ADAR1^ was largely cytoplasmic (Figure 4b; i–iii). In the presence of arsenite, both TIAR and GFP-Zα^ADAR1^ co-localized to stress granules (Figure 4b; iv–vi). These data therefore confirmed that the Zα^ADAR1^ domain was sufficient to direct localization of GFP to stress granules. Moreover, equivalent observations were made when the Zα^ADAR1^ domain was fused to mCherry (data not shown).

To corroborate our findings, we went on to create additional Flag-tagged constructs where the Zα^ADAR1^ domain was placed either downstream or upstream of the coding region for the polypyrimidine tract binding protein 4 (PTB4) (32) (Figure 4c; Flag-PTB4-Zα^ADAR1^ and Flag-Zα^ADAR1^-PTB4, respectively). A construct encoding Flag-PTB4 was used as a control (Figure 4c). HeLa cells were transfected with the various PTB4 expression constructs, and after 24 h, they were cultured in the absence or presence of arsenite. Immunoblotting was used to confirm equal expression (Figure 4d). Cells were then fixed and stained with antibodies specific for TIAR (red) or Flag (green). In untreated cells, Flag-PTB4 exclusively localized to the nucleus (Figure 4e; ii and iii). In contrast, addition of the Zα^ADAR1^ domain either downstream or upstream of PTB4 (Flag-PTB4-Zα^ADAR1^ or Flag-Zα^ADAR1^-PTB4, respectively) resulted in both nuclear and cytoplasmic localization (Figure 4f and g; ii and iii). This altered localization of PTB4 was likely to result from inclusion of the NES within the Zα^ADAR1^ domain. In arsenite-treated cells, Flag-PTB4 remained in the nucleus while TIAR localized to stress granules (Figure 4e; iv–vi). In contrast, Flag-PTB4-Zα^ADAR1^ and Flag-Zα^ADAR1^-PTB4 efficiently co-localized with TIAR in stress granules following arsenite treatment (Figure 4f, g; iv–vi, and h). The Zα^ADAR1^ domain is thus sufficient to direct localization of PTB4 to stress granules. The observation that both Flag-PTB4-Zα^ADAR1^ and Flag-Zα^ADAR1^-PTB4 localized to stress granules additionally revealed that this function of the Zα^ADAR1^ domain is independent of its relative position. Moreover, similar observations were made when the Zα domain was fused to the coding region for the RNA-binding protein Raver1 (31) (data not shown). These data together therefore demonstrated that the Zα domain from ADAR1^p150^ was sufficient to direct localization of various proteins to cytoplasmic stress granules.

### The Zα domain is sufficient for localization to stress granules induced by poly(IC)

The Zα domain from ADAR1^p150^ was sufficient for localization of various proteins to arsenite-induced stress granules in HeLa cells (Figures 2–4). We next asked whether the Zα^ADAR1^ domain was sufficient for localization to stress granules induced by transfection of long dsRNA [poly(IC)], which triggers an interferon response. In this case, long dsRNA typically induces stress granules via activation of PKR (protein kinase R), which phosphorylates eIF-2α and thereby inhibits translation (37). We have previously shown that ADAR1^p150^ localizes to stress granules that assemble in response to long dsRNA (24).

**Figure 5.**
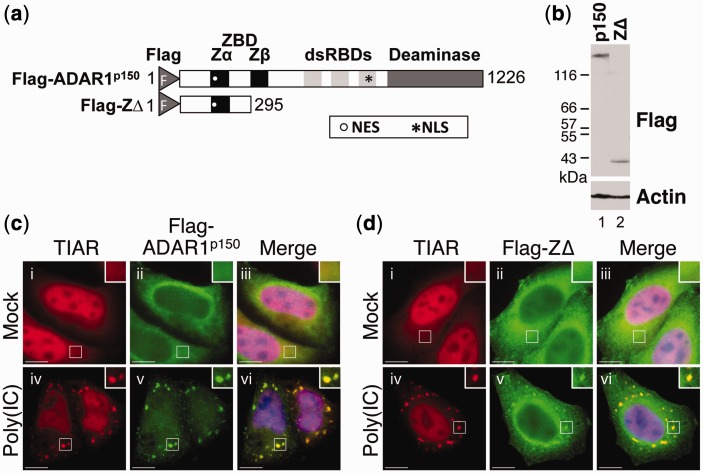
The Zα domain of ADAR1^p150^ localizes to stress granules induced by poly(IC). (**a**) A schematic diagram of Flag-ADAR1^p150^ and Flag-ZΔ. The position of the NES and NLS in each construct is shown as a white circle and a black asterisk, respectively. The Flag epitope tag (F), the Z-DNA-binding domains (ZBD; Zα and Zβ), dsRBDs and deaminase domain are indicated. (**b**) HeLa cells were transfected with the expression constructs Flag-ADAR1^p150^ (p150) and Flag-ZΔ (ZΔ), and lysates were prepared after 24 h. Immunoblotting with a Flag antibody was used to analyze expression. Actin was a loading control. (**c** and **d**) HeLa cells were transiently transfected with expression vectors for Flag-ADAR1^p150^ (c) and Flag-ZΔ (d). After 24 h, the cells were either mock transfected (i–iii) or transfected with poly(IC) (iv–vi) before processing for visualization of TIAR (red; i and iv) and Flag-tagged proteins (green; ii and v) using fluorescence microscopy DAPI staining is in blue (Merge; iii and vi). Bar = 10 μm.

Expression constructs encoding either Flag-ADAR1^p150^ or Flag-ZΔ, which comprised the N-terminal region of ADAR1^p150^ and thus contained the Zα^ADAR1^ domain (Figure 5a), were used to transfect HeLa cells. Immunoblotting using a Flag antibody confirmed that expression of Flag-ADAR1^p150^ and Flag-ZΔ was comparable (Figure 5b). After 24 h, the HeLa cells were either mock-transfected or transfected with poly(IC) to induce an interferon response, as described previously (24). After a further 7 h, the cells were fixed and stained for visualization of TIAR (red) or Flag-tagged proteins (green) using fluorescence microscopy. In mock-transfected cells, both Flag-ADAR1^p150^ and Flag-ZΔ were largely cytoplasmic (Figure 5c and d; ii and iii). Following transfection of cells with poly(IC), both Flag-ADAR1^p150^ and Flag-ZΔ co-localized with TIAR in stress granules (Figure 5c and d; iv–vi). These data therefore demonstrated that the Zα domain from ADAR1^p150^ was sufficient for localization to stress granules induced by poly(IC).

### Zα domains from ZBP1 and E3L proteins also result in stress granule localization

The Zα domain from ADAR1^p150^ was sufficient to target various proteins to stress granules (Figures 4 and 5). We went on to investigate whether the Zα domain found in other Z-DNA binding proteins (ZBP1 and E3L) also gives rise to stress granule localization. Despite the fact that the Zα domains from ADAR1^p150^, ZBP1 and E3L share only ∼25% sequence identity, they are all functional Z-DNA-binding domains (14,22,23).

**Figure 6.**
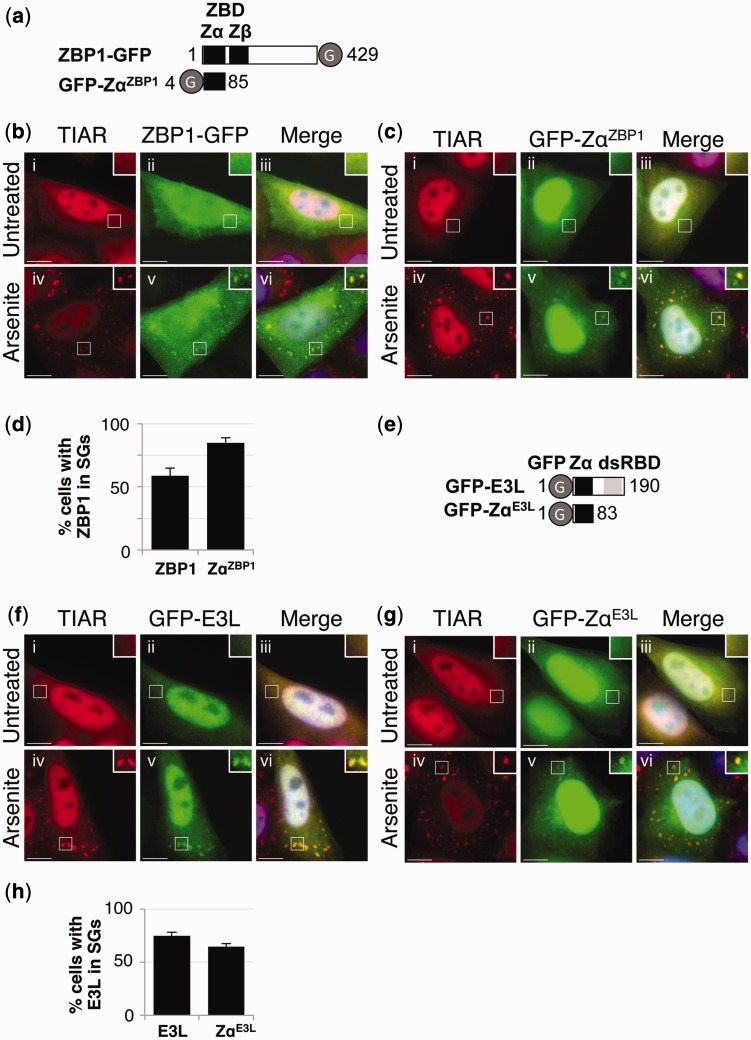
The Zα domain from ZBP1 and E3L localizes to stress granules. (**a**) A schematic diagram of ZBP1-GFP and GFP-Zα^ZBP1^. GFP (G) and the Z-DNA-binding domains (ZBD; Zα and Zβ) are indicated. (**b** and **c**) HeLa cells were transiently transfected with expression vectors for ZBP1-GFP (b) and GFP-Zα^ZBP1^ (c). After 24 h, the cells were cultured in the absence (i–iii) or presence of arsenite (iv–vi) before processing for visualization of TIAR (red; i and iv) and GFP-tagged proteins (green; ii and v) using fluorescence microscopy. DAPI staining is in blue (Merge; iii and vi). Bar = 10 μm. (**d**) TIAR was used as a marker to identify stress granule-containing cells, and the proportion of cells with stress granules positive for ZBP1-GFP (ZBP1) or GFP-Zα^ZBP1^ (Zα^ZBP1^) was then determined. Error bars are mean ± SD (*n* = 3). (**e**) A schematic diagram of GFP-E3L and GFP-Zα^E3L^. GFP (G) and the Zα domain are indicated. (**f** and **g**) HeLa cells were transiently transfected with an expression vector for GFP-E3L (f) or GFP-Zα^E3L^ (g), and after 24 h, the cells were cultured in the absence (i–iii) or presence of arsenite (iv–vi) before processing for visualization of TIAR (red; i and iv) and GFP-tagged proteins (green; ii and v) using fluorescence microscopy. DAPI staining is in blue (Merge; iii and vi). Bar = 10 μm. (**h**) TIAR was used as a marker to identify stress granule-containing cells, and the proportion of cells with stress granules positive for GFP-E3L (E3L) or GFP-Zα^E3L^ (Zα^E3L^) was then determined. Error bars are mean ± SD (*n* ≥ 3).

Expression constructs were initially prepared where GFP was fused to either full-length ZBP1 or the Zα domain from ZBP1 (Zα^ZBP1^) to give ZBP1-GFP and GFP-Zα^ZBP1^, respectively (Figure 6a). The boundaries of the Zα^ZBP1^ domain were as described previously (23). HeLa cells were transfected with ZBP1-GFP or GFP-Zα^ZBP1^, and after 24 h, cells were treated with or without arsenite. Cells were subsequently fixed and processed for visualization of TIAR (red) or GFP-tagged proteins (green) using fluorescent microscopy. In the absence of arsenite treatment, ZBP1-GFP was distributed throughout the cell, whereas GFP-Zα^ZBP1^ was enriched in the nucleus (Figure 6b and c; ii and iii). In the presence of arsenite, both ZBP1-GFP and GFP-Zα^ZBP1^ co-localized with TIAR in stress granules (Figure 6b, c; iv–vi, and d). The relatively weak appearance of the stress granules observed with GFP-Zα^ZBP1^ was due to the strong nuclear signal. Although localization of GFP-Zα^ZBP1^ to stress granules appeared to be slightly better than the full-length protein (ZBP1-GFP; Figure 6d), this may simply reflect a difference in expression. Localization of full-length ZBP1-GFP in stress granules was consistent with previous data (23). However, localization of the Zα^ZBP1^ domain alone to stress granules had not previously been shown. This observation was nevertheless consistent with previous findings where deletion of the Zα^ZBP1^ domain abolished stress granule localization (23). Our data together now confirmed that the Zα^ZBP1^ domain was sufficient to localize GFP to stress granules following oxidative stress.

We next investigated whether the Zα domain from the vaccinia virus E3L protein (Zα^E3L^) also resulted in stress granule localization following arsenite treatment. Expression vectors were prepared where the coding region for GFP was inserted upstream of the sequence for either full-length E3L or Zα^E3L^ (GFP-E3L and GFP-Zα^E3L^, respectively; Figure 6e). GFP-E3L and GFP-Zα^E3L^ were subsequently used to transfect HeLa cells, and after 24 h, the cells were cultured in the absence or presence of arsenite, then fixed and processed for visualization of TIAR (red) and GFP-tagged proteins (green) using fluorescence microscopy. In the absence of arsenite, GFP-E3L and GFP-Zα^E3L^ were found in both the nucleus and cytoplasm, although they were enriched in the nucleus (Figure 6f and g; ii and iii). In the presence of arsenite, both GFP-E3L and GFP-Zα^E3L^ co-localized with TIAR in stress granules (Figure 6f, g; iv–vi, and h). These data therefore confirmed that the Zα^E3L^ domain was also sufficient for localization to stress granules.

These data together enabled us to conclude that all of the Zα-containing proteins tested (ADAR1^p150^, ZBP1 and E3L) localize to stress granules, and that the Zα domain was sufficient for their localization.

### Amino acids required for Z-RNA binding are required for stress granule localization

We have convincingly demonstrated that the presence of a Zα domain is sufficient for localization of proteins to cytoplasmic stress granules (Figures 2–5). We now wanted to determine whether this function of the Zα domain depended on its ability to bind either Z-RNA or Z-DNA.

Structural analyses previously identified a number of highly conserved amino acid residues in the Zα^ADAR1^ domain that are critical for interacting with both Z-DNA and Z-RNA (13,38). Moreover, co-crystal structures of Zα^ZBP1^ and Zα^E3L^ have shown that similar residues are likewise important for Z-DNA binding in other Zα domains (14,17). Mutagenesis further highlighted the importance of these amino acid residues for Z-DNA binding, as exemplified by various studies (22,23,39). We next tested whether mutation of key residues in the Zα^ADAR1^ domain that are essential for Z-DNA/Z-RNA binding would interfere with localization to stress granules.

**Figure 7.**
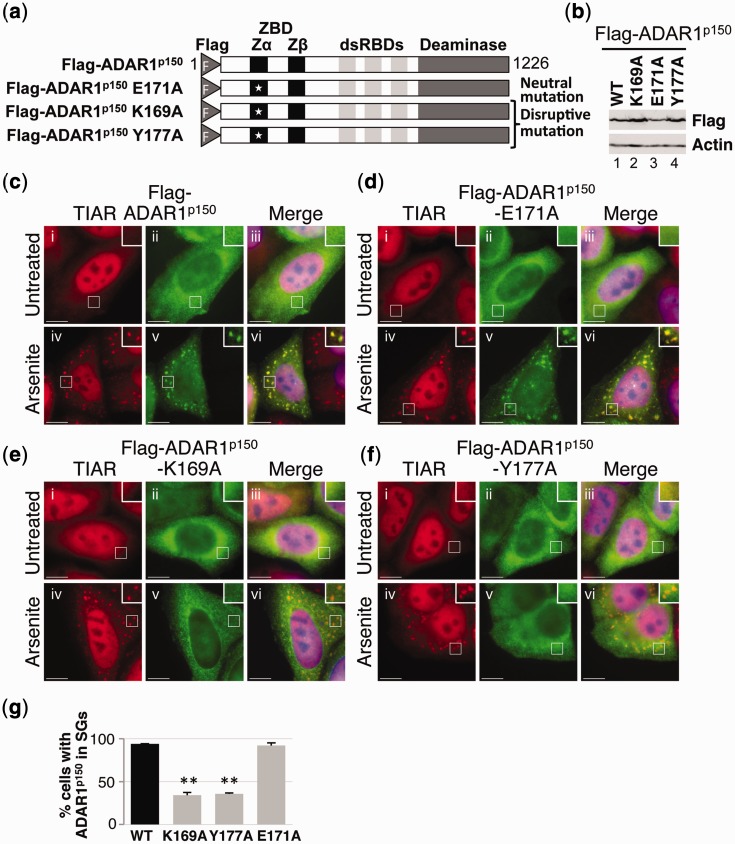
Z-RNA-binding residues are required for localization to stress granules. (**a**) A schematic diagram of Flag-ADAR1^p150^, Flag-ADAR1^p150^ K169A, Flag-ADAR1^p150^ Y177A and Flag-ADAR1^p150^ E171A. White stars indicate the positions of the point mutations. The Flag epitope tag (F), Z-DNA-binding domains (ZBD; Zα and Zβ), dsRBDs and deaminase domain are indicated. (**b**) HeLa cells were transfected with expression vectors for wild-type Flag-ADAR1^p150^ (WT), Flag-ADAR1^p150^ K169A (K169A) Flag-ADAR1^p150^ E171A (E171A) and Flag-ADAR1^p150^ Y177A (Y177A), and lysates were prepared after 24 h. Immunoblotting with a Flag antibody was used to analyze expression. Actin was a loading control. (**c–f**) HeLa cells were transiently transfected with expression vectors for Flag-ADAR1^p150^ (c), Flag-ADAR1^p150^ E171A (d), Flag-ADAR1^p150^ K169A (e) and Flag-ADAR1^p150^ Y177A (**f**). After 24 h, the cells were cultured in the absence (i–iii) or presence of arsenite (iv–vi) before processing for visualization of TIAR (red; i and iv) or Flag-tagged proteins (green; ii and v) using fluorescence microscopy. DAPI staining is in blue (Merge; iii and vi). Bar = 10 μm. (**g**) TIAR was used as a marker to identify stress granule-containing cells, and the proportion of cells with stress granules positive for wild-type Flag-ADAR1^p150^ (WT) or Flag-ADAR1^p150^ mutants (K169A, Y177A, and E171A) was then determined. *P* ≤ 1 × 10^−3^ (double asterisk). Error bars are mean ± SD (*n* = 3).

Expression constructs were therefore prepared in which single point mutations were introduced into full-length Flag-ADAR1^p150^ to give Flag-ADAR1^p150^ E171A, Flag-ADAR1^p150^ K169A and Flag-ADAR1^p150^ Y177A (Figure 7a). Although mutation of E^171^ was predicted to be a neutral mutation that would not affect nucleic acid binding, mutation of either Lys^169^ or Tyr^177^ was expected to disrupt binding of Flag-ADAR1^p150^ to Z-DNA or Z-RNA (13,39). HeLa cells were transfected with the various ADAR1^p150^ expression constructs, and after 24 h, they were cultured in the absence or presence of arsenite. Immunoblotting with a Flag antibody was used to show that expression was comparable (Figure 7b). Cells were subsequently fixed and stained with antibodies to TIAR (red) and Flag (green) for visualization by fluorescence microscopy. In the absence of arsenite, Flag-ADAR1^p150^, Flag-ADAR1^p150^ E171A, Flag-ADAR1^p150^ K169A and Flag-ADAR1^p150^ Y177A were all predominantly cytoplasmic (Figure 7c–f; ii and iii). Following arsenite treatment, both Flag-ADAR1^p150^ and Flag-ADAR1^p150^ E171A efficiently co-localized with TIAR in stress granules (Figure 7c, d; iv–vi and g). In contrast, the proportion of cells in which either Flag-ADAR1^p150^ K169A or Flag-ADAR1^p150^ Y177A co-localized with TIAR in stress granules was significantly less (Figure 7e, f; iv–vi and g). Moreover, when co-localization in stress granules was observed, the stress granules generally appeared much weaker than those seen with wild-type ADAR1^p150^ (data not shown). These data therefore confirmed that mutation of the amino acid residues necessary for binding to Z-RNA or Z-DNA substantially impaired localization of ADAR1^p150^ to stress granules. Moreover, similar observations were made when the same mutations were introduced into Flag-ZR, Flag-Z and Flag-ZΔ expression constructs (Supplementary Figures S1–S3). In addition, when equivalent mutations were made in the vaccinia virus E3L protein, a reduction in stress granule localization was also observed (Supplementary Figure S4).

These data together therefore allowed us to conclude that binding of ADAR1^p150^ to Z-DNA or Z-RNA is necessary for localization to stress granules. As stress granules are cytoplasmic, it is plausible that Z-RNA is the most likely candidate to fulfill this role. Although many proteins have been shown to localize to stress granules as a result of mRNA binding (25), localization of specific proteins to stress granules via Z-RNA binding represents a novel method of recruitment.

## DISCUSSION

We have conclusively demonstrated that the Zα domain from ADAR1^p150^ is necessary and sufficient for localization of ADAR1^p150^ to cytoplasmic stress granules in HeLa cells following either oxidative stress or transfection of long dsRNA. Furthermore, we have shown that the Zα domain from both ZBP1 and E3L is also able to direct localization to stress granules. Finally, our data suggest that localization of the Zα^ADAR1^ domain to stress granules is dependent on its ability to interact with either Z-RNA or Z-DNA. We have thus identified a novel role for the Z-DNA-binding domains that are found within a limited number of proteins in eukaryotic cells.

We previously speculated that ADAR1^p150^ may localize to cytoplasmic stress granules following editing of cytoplasmic RNA targets, whereby the IU-dsRNA generated could trigger stress granule formation following specific interaction with a stress-granule-like complex (24,33). In this scenario, ADAR1^p150^ would localize to stress granules by virtue of association with the edited dsRNA. However, our data now rule out this possible explanation for how ADAR1^p150^ localizes to stress granules, as localization does not require a functional deaminase domain (Figure 1). Rather, our data suggest that recruitment of ADAR1^p150^ to stress granules depends on its interaction with Z-RNA within stress granules. Although we are unable to rule out the possibility that ADAR1^p150^ also interacts with Z-DNA, this seems an unlikely scenario due to its cytoplasmic location. The idea that Z-RNA exists in stress granules is in keeping with previous studies that showed that Z-RNA is abundant in both the cytoplasm and the nucleolus (40). The idea that ADAR1^p150^ localizes to stress granules by interacting with Z-RNA is intriguing. This scenario represents not only a novel method for localization of proteins to stress granules but also one that will result in rigorous selection of the few proteins that contain a Z-DNA-binding domain (9,41). It is therefore essential to consider the identity of the Z-RNAs that may be present in stress granules.

Various genome-wide studies have been undertaken to identify potential Z-DNA-forming regions (ZDRs) within eukaryotic genomes (19,42,43). Although *in silico* predictions have dominated these studies, one recent study additionally used a Z-DNA-specific probe (Zα^ADAR1^) to obtain direct evidence for the existence of ZDRs in Z-conformation within human cells (19). *In silico* predictions alone suggested that ZDRs occur preferentially within sequences in close proximity of transcription start sites, where the Z-DNA would be stabilized by negative supercoiling that occurs in the wake of the translocating RNA polymerase (15,42,43). In contrast, when a Z-DNA-specific probe was used in conjunction with computational analyses, of the 186 ZDR hotspots identified, only two mapped to transcription start sites (19). Instead, many of the hotspots identified were found within tandem repeat sequences, including ALR/alpha satellite sequences and *Alu* subfamilies. The remaining ZDRs were predicted within introns and exons, consistent with previous data (43). Although these data focused on putative Z-DNA-forming sequences, it is likely that Z-RNA occurs within equivalent sequences. We therefore speculate that Z-RNA exists within the population of cellular RNAs sequestered within stress granules, which may include *Alu* sequences (25,26,44). It is likely that similar sequences exist within other mammalian genomes that also have the potential to form Z-DNA, including those found within introns and exons, as well as alternative tandem repeat sequences. It is therefore likely that Z-RNA would similarly exist in stress granules found in other mammalian cells. As discussed earlier in the text, we anticipate that any Z-RNA found within stress granules will interact specifically with proteins that contain Z-DNA-binding domains, such as ADAR1^p150^, and that this interaction will be essential for their recruitment (Figure 7). Interaction with other stress granule proteins may subsequently contribute to the stable association of ADAR1^p150^ in stress granules (24).

It is interesting to speculate that Z-RNA may form within *Alu* sequences that localize to stress granules in human cells (44). Previous data have shown that the majority of editing occurs within non-coding regions of RNA, particularly within *Alu* repeat sequences (45–48). These observations together lead us to speculate that editing of *Alu* sequences may occur following recruitment of ADAR1^p150^ to stress granules by Z-RNA binding. This idea is in keeping with a previous study that showed that editing of an RNA target was markedly enhanced when it contained a sequence that favored Z-RNA formation (11). Editing of the *Alu* sequence would thus give rise to IU-dsRNA, which would in turn suppress the induction of interferon and apoptosis triggered by stress (49). Cell survival would thus be enhanced following recruitment of ADAR1^p150^ to stress granules.

We have shown that full-length ADAR1^p150^, ZBP1 and E3L localize to stress granules (Figures 1–3 and 5–7). Localization of ZBP1 to stress granules is consistent with previous findings (23). However, we have gone further to show that the minimal Zα domain from ADAR1^p150^ (Zα^ADAR1^), ZBP1 (Zα^ZBP1^) or E3L (Zα^E3L^) fused to other proteins is sufficient to direct their localization to stress granules (Figures 4 and 6). The observation that the Zα^ZBP1^ domain is sufficient for stress granule localization is in keeping with previous findings where deletion of Zα^ZBP1^ from ZBP1 abolished stress granule localization (23). It is intriguing that each of the Z-DNA-binding domain-containing proteins plays a role in the immune response in mammalian cells. As mentioned earlier in the text, ADAR1^p150^ and ZBP1 are upregulated by interferon and have been implicated in host response mechanisms (16,21). Moreover, ADAR1^p150^ appears to play both antiviral and pro-viral roles in mammalian cells depending on the virus-host combination (50). The E3L protein from vaccinia virus antagonizes the actions of interferon and is thus essential for viral pathogenicity (22). Moreover, this observation depends on the presence of a functional Zα domain within E3L. Previous studies have postulated that the Zα domain of E3L may compete with host proteins such as ADAR1^p150^ and ZBP1 for binding nucleic acids in the Z-conformation (23). In light of our current data, we can now extend this hypothesis to propose that these proteins will compete for binding Z-RNA present in stress granules, which may in turn interfere with the role they play during the immune response in mammalian cells. In addition, co-localization of E3L and ADAR1 is likely to antagonize the enzymatic function of ADAR1^p150^ (41). We now intend to identify the Z-RNAs that bind ADAR1^p150^, ZBP1 and E3L in stress granules to understand the potential interplay between these proteins and how this impacts on the immune response.

The data we have described herein have identified a novel role for the Zα domain found in ADAR1^p150^ and other proteins that contain Z-DNA-binding domains. We have demonstrated that the Zα domain is necessary and sufficient for localization to cytoplasmic stress granules. This unexpected observation raises some interesting questions as to why Z-RNA should be used to localize proteins containing Z-DNA-binding domains to stress granules. Further studies will be therefore be key to fully understand how these proteins contribute to the immune response in mammalian cells. Nevertheless, our findings describe a hitherto unknown function for Z-DNA-binding domains.

## SUPPLEMENTARY DATA

Supplementary Data are available at NAR Online.

## FUNDING

Biotechnology and Biological Sciences Research Council [BB/F018347/1 to A.D.J.S.]. Funding for open access charge: University of Cambridge.

*Conflict of interest statement*. None declared.

## Supplementary Material

Supplementary Data
