# Supplementary material for: Proteins that contain a functional Z-DNA-binding domain localize to cytoplasmic stress granules
Source: Nucleic Acids Res. 2013 Aug 27;41(21):9786–99. doi: 10.1093/nar/gkt750 (PMC3834823; doi:10.1093/nar/gkt750)
Supplement: Supplementary Data [file supp_41_21_9786__index.html]

Proteins that contain a functional Z-DNA-binding domain localize to cytoplasmic stress granules — Proteins that contain a functional Z-DNA-binding domain localize to cytoplasmic stress granules — Supplementary Data 

# Proteins that contain a functional Z-DNA-binding domain localize to cytoplasmic stress granules

## Supplementary Data

files

**Files in this Data Supplement:**

- Supplementary Data - pdf file
