# Supplementary material for: Proteins that contain a functional Z-DNA-binding domain localize to cytoplasmic stress granules
Source: Nucleic Acids Res. 2013 Aug 27;41(21):9786–99. doi: 10.1093/nar/gkt750 (PMC3834823; doi:10.1093/nar/gkt750)
Supplement: Supplementary Data [file supp_gkt750_nar-01748-f-2013-File010.pdf]

SUPPLEMENTARY MATERIALS AND METHODS

Expression constructs

Constructs were prepared using standard cloning procedures (30). For Flag-ZΔ, the ZΔ domain of ADAR1^{p150} (amino acids (aa) 1–295; numbered as for ADAR1 p150) was amplified by PCR using primers containing *Hin*dIII and *Xba*I restriction sites, and inserted into p3xFLAG-CMV™-7.1 (Sigma). The same strategy was used to clone Flag-Z (aa 1–361), and Flag-ZR (aa 1–807). Mutations within Flag-ZΔ, Flag-Z and Flag-ZR (K169A, E171A, Y177A, E912A) were introduced by site directed mutagenesis (Stratagene). For Flag-E3L, full length E3L (aa 1–190) was amplified by PCR using primers containing *Hin*dIII and *Bam*HI restriction sites, and inserted into p3xFLAG-CMV™-7.1 (Sigma). Mutations within Flag-E3L (K40A, E42A, Y48A) were introduced by site directed mutagenesis (Stratagene). All constructs were confirmed by sequencing.

SUPPLEMENTARY FIGURE LEGENDS

Supplementary Figure 1. Z-RNA binding residues are required for localization to stress granules. (**a**) A schematic diagram of Flag-ZΔ, Flag-ZΔ K169A, Flag-ZΔ Y177A and Flag-ZΔ E171A. White stars indicate the positions of the point mutations. The Flag epitope tag (F), Z-DNA binding domains (ZBD; Zβ and Zα), dsRNA binding domains (dsRBDs) and deaminase domain are indicated. (**b**) HeLa cells were transfected with expression vectors for wild type (WT) Flag-ZΔ, Flag-ZΔ E171A, Flag-ZΔ K169A and Flag-ZΔ Y177A, and lysates prepared after 24 h. Immunoblotting was used to analyze expression. Actin was a loading control. (**c–f**) HeLa cells

were transiently transfected with expression vectors for Flag-ZΔ (**c**), Flag-ZΔ E171A (**d**), Flag-ZΔ K169A (**e**) and Flag-ZΔ Y177A (**f**). After 24 h the cells were cultured in the absence (i–iii) or presence of arsenite (iv–vi) before processing for visualization of TIAR (red; i, iv) or Flag-tagged proteins (green; ii, v) using fluorescence microscopy. DAPI staining is in blue (Merge; iii, vi). Bar=10 μm. (**g**) TIAR was used as a marker to identify stress granule-containing cells, and the proportion of cells with stress granules positive for Flag-ZΔ (WT) or Flag-ZΔ mutants (K169A, Y177A, E171A) was then determined. *P* values were $\leq 2\times10^{-3}$ (**). Error bars are mean ± s.d (n=3).

Supplementary Figure 2. Z-RNA binding residues are required for localization to stress granules. (**a**) A schematic diagram of Flag-Z, Flag-Z K169A, Flag-Z Y177A and Flag-Z E171A. White stars indicate the positions of the point mutations. The Flag epitope tag (F), Z-DNA binding domains (ZBD; Zβ and Zα), dsRNA binding domains (dsRBDs) and deaminase domain are indicated. (**b**) HeLa cells were transiently transfected with expression vectors for wild type (WT) Flag-Z, Flag-Z E171A, Flag-Z K169A and Flag-Z Y177A, and lysates prepared after 24 h. Immunoblotting was used to analyze expression. Actin was a loading control. (**c–f**) HeLa cells were transiently transfected with expression vectors for Flag-Z (**c**), Flag-Z E171A (**d**), Flag-Z K169A (**e**) and Flag-Z Y177A (**f**). After 24 h the cells were cultured in the absence (i–iii) or presence of arsenite (iv–vi) before processing for visualization of TIAR (red; i, iv) or Flag-tagged proteins (green; ii, v) using fluorescence microscopy. DAPI staining is in blue (Merge; iii, vi). Bar=10 μm. (**g**) TIAR was used as a marker to identify stress granule-containing cells, and

the proportion of cells with stress granules positive for Flag-Z (WT) or Flag-Z mutants (K169A, Y177A, E171A) was then determined. *P* values were ≤1×10^{-3} (**). Error bars are mean ± s.d (n=3).

Supplementary Figure 3. Z-RNA binding residues are required for localization to stress granules. (**a**) A schematic diagram of Flag-ZR, Flag-ZR K169A, Flag-ZR Y177A and Flag-ZR E171A. White stars indicate the position of the point mutation. The Flag epitope tag (F), Z-DNA binding domains (ZBD; Zβ and Zα), dsRNA binding domains (dsRBDs) and deaminase domain are indicated. (**b**) HeLa cells were transiently transfected with expression vectors for wild type (WT) Flag-ZR, Flag-ZR E171A, Flag-ZR K169A and Flag-ZR Y177A, and lysates prepared after 24 h. Immunoblotting was used to analyze expression. Actin was a loading control. (**c–f**) HeLa cells were transiently transfected with expression vectors for Flag-ZR (**c**), Flag-ZR E171A (**d**), Flag-ZR K169A (**e**) and Flag-ZR Y177A (**f**). After 24 h the cells were cultured in the absence (i–iii) or presence of arsenite (iv–vi) before processing for visualization of TIAR (red; i, iv) or Flag-tagged proteins (green; ii, v) using fluorescence microscopy. DAPI staining is in blue (Merge; iii, vi). Bar=10 µm. (**g**) TIAR was used as a marker to identify stress granule-containing cells, and the proportion of cells with stress granules positive for Flag-ZR (WT) or Flag-ZR mutants (K169A, Y177A, E171A) was then determined. *P* values were ≤2×10^{-3} (**). Error bars are mean ± s.d (n=3).

Supplementary Figure 4. Z-RNA binding residues are required for localization to stress granules. (**a**) A schematic diagram of Flag-E3L, Flag-

E3L E42A, Flag-E3L K40A and Flag-E3L Y48A. White stars indicate the position of the point mutation. The Flag epitope tag (F), Zα domain and dsRNA binding domain (dsRBDs) are indicated. (**b–e**) HeLa cells were transiently transfected with expression vectors for Flag-E3L (**b**), Flag-E3L E42A (**c**), Flag-E3L K40A (**d**) and Flag-E3L Y48A (**e**). After 24 h the cells were cultured in the absence (i–iii) or presence of arsenite (iv–vi) before processing for visualization of TIAR (red; i, iv) or Flag-tagged proteins (green; ii, v) using fluorescence microscopy. DAPI staining is in blue (Merge; iii, vi). Bar=10 μm. (**f**) TIAR was used as a marker to identify stress granule-containing cells, and the proportion of cells with stress granules positive for Flag-E3L (WT) or Flag-E3L mutants (K40A, Y48A, E42A) was then determined. *P* values were $<1\times10^{-4}$ (**). Error bars are mean ± s.d (n=6).

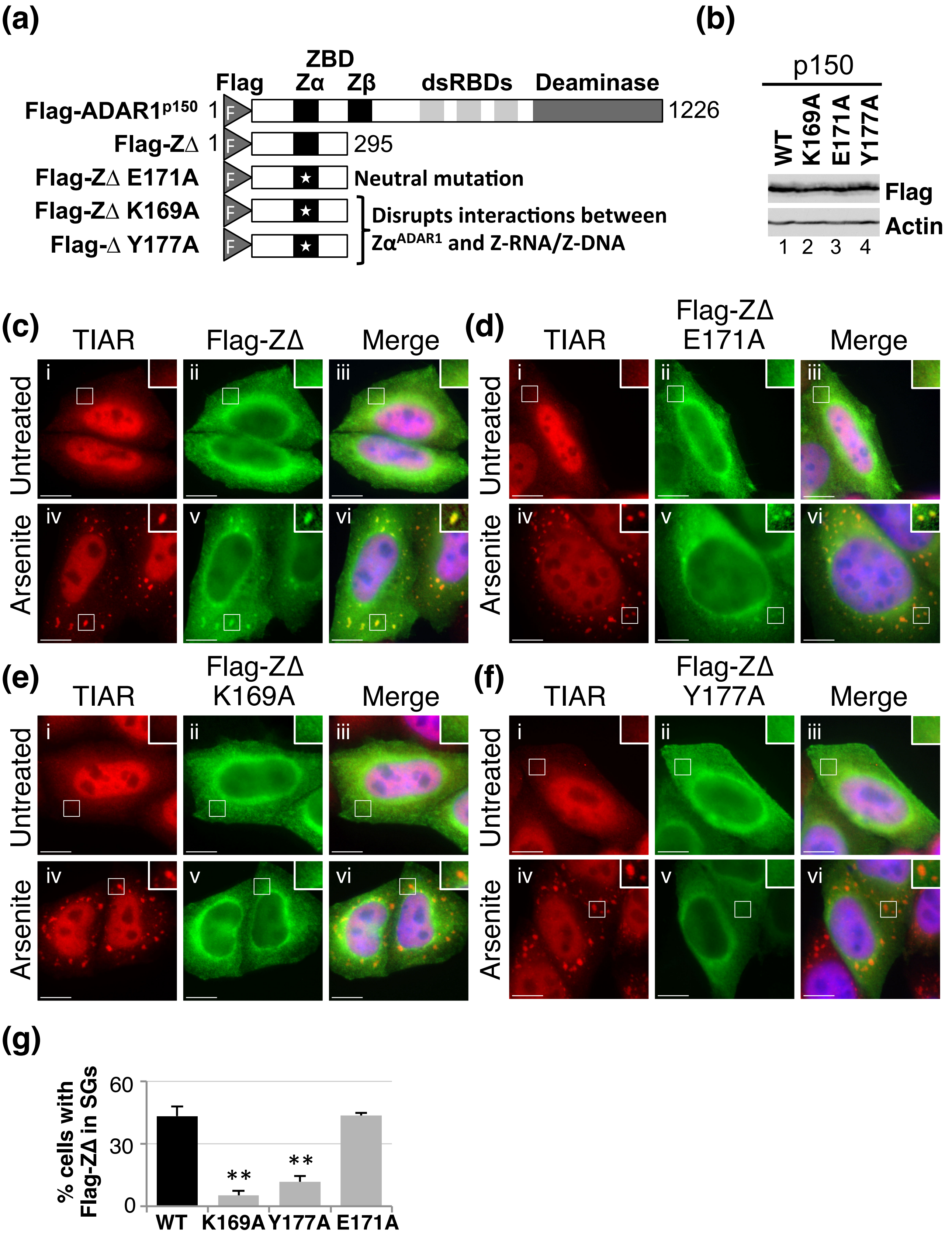

Supplementary Figure S1

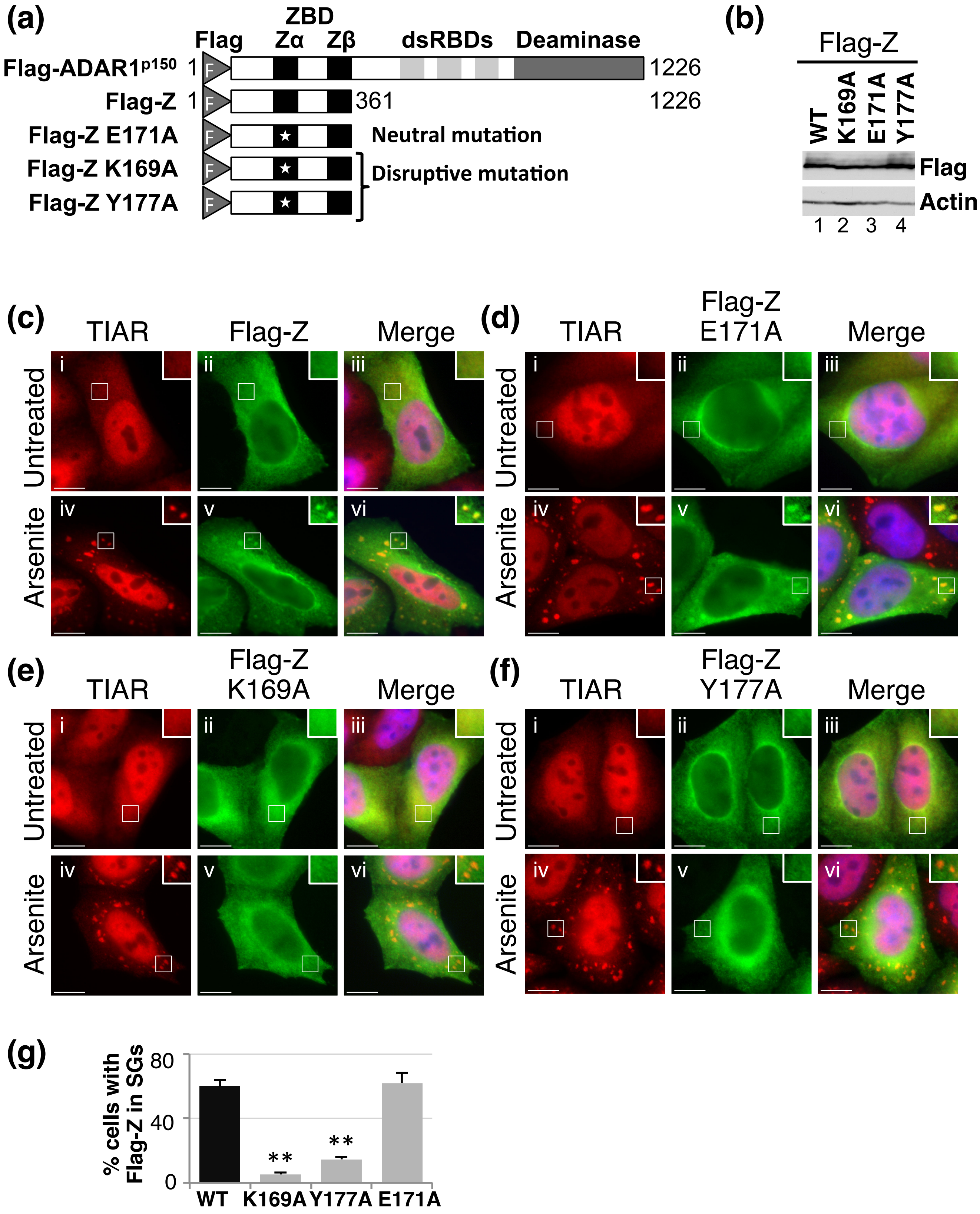

Ng et al

Supplementary Figure S2

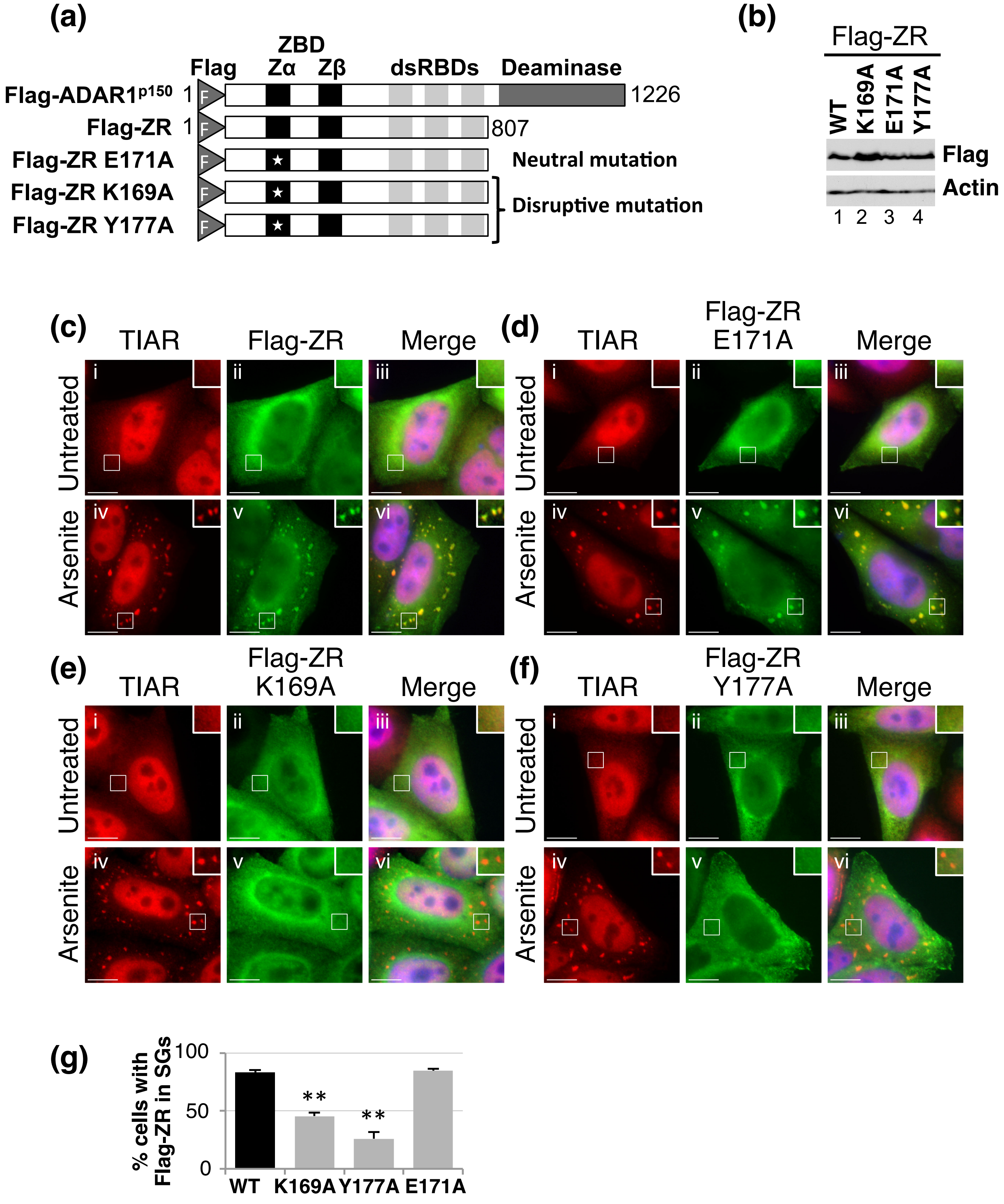

Ng et al

Supplementary Figure S3

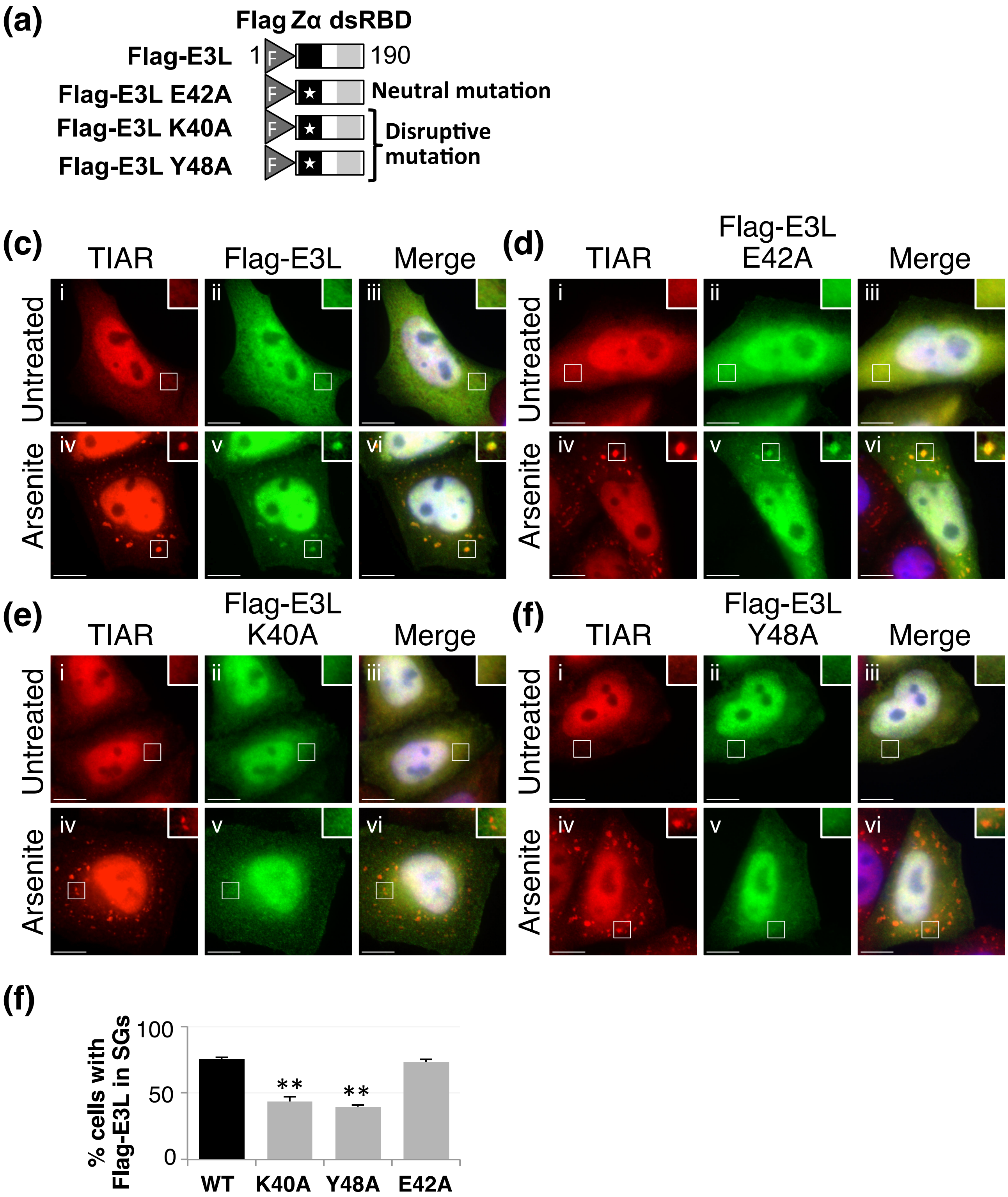

Ng et al

Supplementary Figure S4
